# Fibrolamellar Carcinoma of The Liver — A Case Report From Japan and a Review of The Literature

**DOI:** 10.1155/1988/12681

**Published:** 1988

**Authors:** Hitoshi Kohno, Naofumi Nagasue, Hiroyuki Taniura, Teruhisa Nakamura, Sabro Nagaoka

**Affiliations:** 1 Second Department of Surgery and Clinical Department of Pathology Shimane Medical University Izumo 693 Japan; 2 Second Department of Surgery Shimane Medical University Izumo 693 Japan

## Abstract

The first Japanese case of fibrolamellar carcinoma (FLC) of the liver is described. The 17-year-old Japanese male affected died of the disease 25 months after palliative operation. The diagnosis of FLC had not been confirmed before autopsy. Pre-operative diagnosis of FLC is important to surgeons. Acccording to the literature, FLC is rare in Asia. The endemic discrepancy may suggest that the carcinogenic factors are different from those of general hepatocellular carcinoma.

## INTRODUCTION

Fibrolamellar carcinoma (FLC) of the liver has been widely recognized as a subtype of hepatocellular carcinoma (HCC) since 1980[1,2]. We herein report on a Japanese teenager affected by FLC.

## CASE REPORT

A 17-year-old Japanese male underwent a laparotomy under pre-operative diagnosis of choledocholithiasis at Shimane Medical University on 27 December 1980. The choledochotomy revealed yellow-greenish emboli in the bile ducts. The histologies of both the emboli and the biopsied lymphnodes indicated HCCs (Figure 1). But it was not suggested that this HCC was FLC at that moment. Serum level of alpha-fetoprotein was below 5 ng/ml and that of carcino-embryonic antigen below 2.5 ng/ml. The patient was negative for both hepatitis B surface antigen and antibody. The second laparotomy revealed the hepatoma (8 × 8 × 8 cm) at the right hepatic lobe. Although hepatic-arteries ligation and infusion chemotherapy (5.25 g of 5-fluorouracil and 100 mg of Doxorubicin) were performed, the patient died on 9 February 1983. It was realized at the postmortem examination that the HCC of this patient was a special subtype.

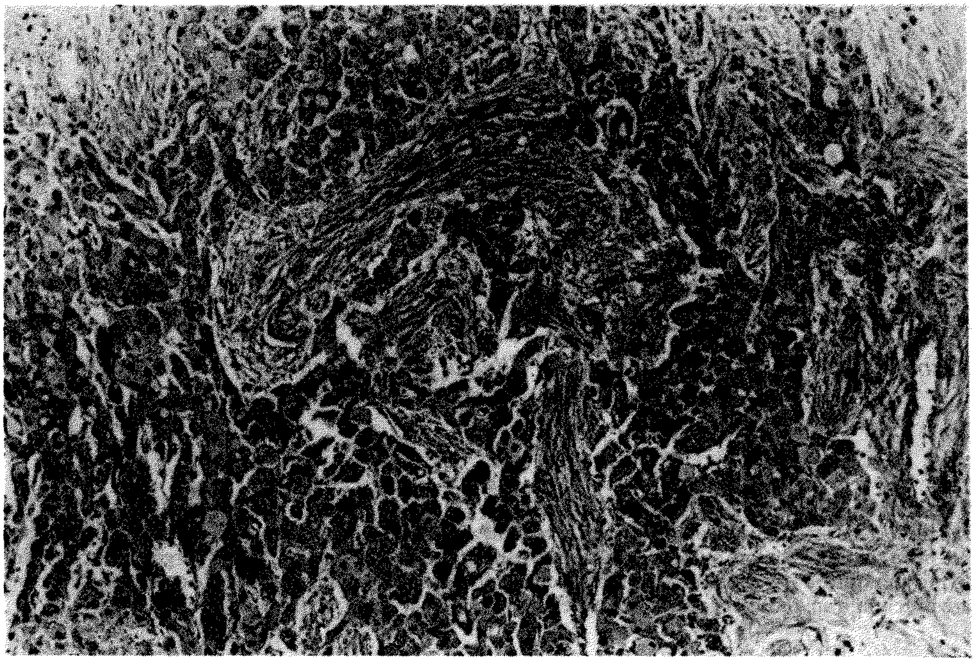

**Figure 1** Histology of the tumor in the common hepatic duct shows lamellar fibrosis and polygonal malignant hepatocytes (H & E 25).

## DISCUSSION

In this case, the diagnosis of FLC was not made before autopsy. Although FLC has some diagnostic characteristics[1–5], it seems difficult to make a definitive diagnosis before surgery in most patients[6]. Even so, such diagnosis may be necessary for surgeons to perform aggressive hepatic resection, since FLC can promise a relatively favorable prognosis after resection. This point has been stressed recently by Dawson[7]. Furthermore, Calne[8] and Starzl *et al.*[9] have claimed that FLC is also a better candidate for liver transplantation.

Incidentally, the patient also had tumor invasion of bile ducts. It is worthwhile to note that FLC, like general HCC, develops intraductal tumor growth in some patients[10].

As far as we know from the literature, there have been 143 cases of FLC (Table 1)[1–6, 9–32]. In Asia, where HCC is more frequent than in the West, there are only three cases, including the present case. The carcinogenic factors of FLC might be different from those of general HCC. Perhaps a world-wide survey is necessary to clarify this.

*Acknowledgements*

The authors thank Sir Roy Y. Calne for his encouragement and Prof. K. Okuda for confirming the histology.

**Table 1** Endemic aspects of fibrolamellar carcinoma in the literature[a].

| *Area* | *References* | *Race of patient* | | | |
|---|---|---|---|---|---|
| | | *White* | *Black* | *Yellow* | *Unknown* |
| North America | 1,2,4,6,9[b],10,14–22, 23[b],24–28 | 61 | 6 | 1 | 53 |
| Europe | 3,5,11,29,30 | — | — | — | 19 |
| Asia | 13 | — | — | 1[c] | 2 |

[a] Three patients reported by Jagirdar *et al*[12], three by Stromeyer *et al.*[31] and nine by Goodman and Ishak[32] are omitted to avoid overlap of patients.

[b] Overlap of patients between these two articles is avoided by careful comparison.

[c] Our patient
